# Contamination of Potable Water Distribution Systems by Multiantimicrobial-Resistant Enterohemorrhagic *Escherichia coli*

**DOI:** 10.1289/ehp.10809

**Published:** 2007-12-21

**Authors:** Siya Ram, Poornima Vajpayee, Rishi Shanker

**Affiliations:** Environmental Microbiology Division, Industrial Toxicology Research Centre, Mahatma Gandhi Marg, Lucknow, India

**Keywords:** drinking water, enterohemorrhagic *Escherichia coli*, multiantimicrobial resistant, virulence determinants

## Abstract

**Background:**

The contamination of processed or unprocessed drinking water by fecal coliform bacteria has been reported worldwide. Despite a high incidence of waterborne diseases, entero-hemorrhagic *Escherichia coli* (EHEC) is an underacknowledged pathogen of concern to public health in India. Although the presence of EHEC is recorded in surface water resources of India, drinking water sources are yet to be investigated.

**Objectives:**

The goal of this study was to analyze potable water samples for the presence of virulence determinants of EHEC and to determine the sensitivity of the virulence determinants to antimicrobials.

**Methods:**

We enumerated coliform bacteria in potable water samples collected from six locations in Lucknow, a major city in northern India, using the most probable number method. *E. coli* (*n* = 81), randomly isolated by membrane-filtration technique from four sites, were identified by biochemical characterization. *E. coli* were not detected in samples from two other sites. We screened 15 randomly selected isolates from each site for virulence determinants of EHEC using polymerase chain reaction (PCR). The isolates positive for virulence determinants (*n* = 18) were screened for sensitivity to 15 antimicrobials by the disk diffusion method.

**Results:**

Both *stx1* and *stx2* genes were present in 33.3% of isolates, whereas others possessed either *stx1* (11.1%) or *stx2* (55.6%). *eaeA, hlyA,* and *chuA* genes were present in 100, 23.3, and 16.7% of isolates, respectively. Resistance to multiple antimicrobials was observed in potential EHEC.

**Conclusions:**

The occurrence of multiantimicrobial-resistant EHEC in potable water is an important health concern because of the risk of waterborne outbreaks.

*Escherichia coli*, a normal inhabitant of the gastrointestinal tract of warm-blooded animals, is used as an indicator of water quality. Certain serotypes have been associated with waterborne disease outbreaks and mortality in humans (Bruneau et al. 2004). Shiga toxin–producing *E. coli* (STEC) or enterohemorrhagic *E. coli* (EHEC) are asymptomatic in animals, but human infections may lead to hemorrhagic colitis, hemolytic uremic syndrome, or death (Shelton et al. 2006). Although cattle represent the main reservoir, EHEC is harbored by a wide range of animals and birds (Williams et al. 2006). EHEC causes diseases in humans through production of one or more shiga-like toxins (encoded by *stx1* and *stx2* and their variants), which inhibit protein synthesis of host cells, leading to cell death. Other virulence factors include the *eaeA* gene-encoding intimin, responsible for attaching and effacing lesions, and the *hlyA* gene, which acts as a pore-forming cytolysin on eukaryotic cells. The ingestion of as few as 1–10 EHEC cells may cause illness in humans (Chart 2000; Kuhnert et al. 2000). EHEC contamination of drinking water (processed and unprocessed) has been associated with disease outbreaks (Ashbolt 2004).

In India and some other countries, surface waters from rivers, lakes, and ponds are processed by alum treatment, filtration, and chlorination to be used as drinking water (Clever et al. 2000; Shrivastava et al. 2004). Some recent studies found multiantimicrobial-resistant *E. coli* isolates positive for virulence determinants for EHEC in surface waters that are being used as raw water to supply drinking water (Hamner et al. 2007; Ram and Shanker 2005; Ram et al. 2007). The occurrence of potential EHEC from extensively used source waters is an important health concern because much of India’s population depends on processed or unprocessed surface waters for drinking. Recently, insufficient treatment of surface waters for the drinking water supply, malfunctioning of sewage collection systems, and defective water distribution pipelines have led to contamination of potable water by fecal coliform and other pathogenic bacteria (Babu and Kumar 2002; Bhatta et al. 2007; Shrivastava et al. 2004). However, despite a high incidence of water-borne diseases in India, the potable water supply has never been investigated for the presence of specific pathotypes of diarrheagenic *E. coli* including EHEC. In India, EHEC has been underacknowledged as far as public health is concerned (Hamner et al. 2007), and thus no report has been published on the presence of multiantimicrobial-resistant *E. coli* exhibiting virulence determinants specific to EHEC in potable water in India. In this article, we report on the occurrence of multiantimicrobial-resistant *E. coli* harboring virulence markers of EHEC in potable water samples collected from the drinking water distribution systems of northern India.

## Materials and Methods

### Sample collection and quantitative enumeration of coliform population

**Figure 1:**
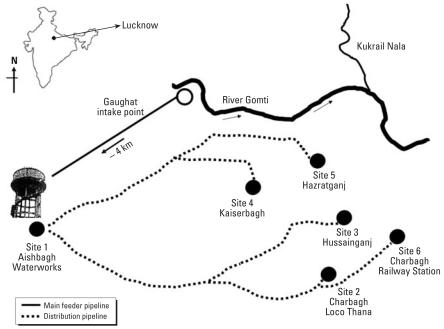
Schematic representation of location of water treatment plant (Lucknow Jal Sansthan, Aishbagh), distribution pipelines, and the six sites of potable water sampling.

The River Gomti passes through Lucknow, India, and is the main source of drinking water for the city. Water is pumped from the river at Gaughat, which is outside the city, and is sent through a pipeline to Lucknow Jal Sansthan, Aishbagh, 4 km away (Figure 1), where the water is purified by alum treatment, filtration, and chlorination before being released into the drinking water supply (Shrivastava et al. 2004). To test the possibility of the contamination of potable waters by EHEC due to defective water distribution systems and insufficient treatment during production, we collected water samples (1 L) in triplicate for isolation of *E. coli* and quantitative enumeration of the coliform population (by the multiple-tube fermentation technique) at six sites: site 1, Aishbagh Waterworks (before water enters the distribution system); site 2, Charbagh Loco Thana; site 3, Hussainganj; site 4, Kaiserbagh (water-distribution pipeline that neither percolated nor ran along open drainage); site 5, Hazaratganj; site 6, Charbagh Railway Station (pipeline that percolated and ran along open drainage) (Figure 1). All samples were collected on the same day in densely populated areas across the urban boundaries of Lucknow (latitude, 26.28 N; longitude, 80.24 E; altitude, 126 m). The water samples were collected into sterile glass bottles, stored on ice, and transported to the laboratory for analyses within 6 hr (American Public Health Association 1998). The sites were numbered in order of the sample collection.

### *Isolation and identification of* E. coli

*E. coli* from water samples was isolated and confirmed to be *E. coli* as described by Ram et al. (2007). A portion of each water sample (100 mL) from each site was filtered in triplicate through a membrane filter (cellulose nitrate filter of 0.45 μm pore size). Each membrane filter was aseptically removed by sterile forceps, cut into four pieces, placed in a 25-mL Erlenmeyer flask containing 10 mL MacConkey broth, and incubated at 35 ± 1°C for 2–4 hr at 220 rpm on a rotary shaker (INNOVA 4230; New Brunswick Scientific, Edison, NJ, USA). A loopful of culture from MacConkey broth tubes was then streaked on Levine EMB (eosin methylene blue) agar plates and incubated overnight at 35 ± 1°C. For further study, we randomly selected 25 blue-black colonies (presumptive *E. coli*) with metallic sheen growing on EMB agar plates from each site (sites 2, 3, 5, and 6). These isolates were screened using indole production, methyl red, Voges-Proskauer, and Simmons citrate tests. The isolates (*n* = 81) confirmed as *E. coli* were maintained at −70°C in Luria Bertani broth supplemented with 15% (vol/vol) glycerol.

### Detection of virulence genes specific to EHEC

We screened 15 randomly selected *E. coli* isolates from each site (sites 2, 3, 5, and 6; a total of 60 isolates) for the presence of virulence genes (*stx1, stx2, eaeA, hlyA, and chuA*) specific to enterohemorrhagic *E. coli* using primers (Table 1) and cyclic conditions described by Ram et al. (2007). In brief, a typical 50-μL polymerase chain reaction (PCR) assay mixture contained 5 μL 10× PCR buffer, μ μL 25 mM magnesium chloride, 1 μL 10 mM dNTP (deoxyribonucleotide triphosphate), 2 μL genomic DNA (30–50 ng) or 5 μL total DNA (lysed cell suspension), 1 μL Taq polymerase (1 unit/μL), and 1 μL of each primer (10 pmol). PCR amplifications were carried out for 35 cycles using the following temperature programs (varying with the type of gene): *stx1*, 94°C for 1 min, 48°C for 1 min, and 72°C for 1 min; *stx2*, 94°C for 1 min, 45°C for 1 min, and 72°C for 1 min; *eae A,* 94°C for 1 min, 44°C for 1 min, and 72°C 1 min; *hlyA*, 94°C for 1 min, 50°C for 1 min, and 72°C 1 min; *chuA,* 94°C for 1 min, 50°C for 1 min, and 72°C for 1 min; *LT1* and *ST1*, 94°C for 1 min, 49°C for 1 min, and 72°C for 1 min. For each gene, the initial denaturation was at 94°C for 4 min, and a final extension was carried out at 72°C for 7 min (iCycler; Bio-Rad, Hercules, CA, USA). PCR-amplified products were resolved on 1.5% agarose gels containing ethidium bromide (0.5 μg/mL) at 3.5 V/cm, and were visualized and recorded using a ChemiImager 4400 gel documentation system (Alpha Innotech Corp., San Leandro, CA, USA). We used the following positive controls: *E. coli* MTCC-723 (Microbial Type Culture Collection, IMTECH, Chandigarh, India) for *LT1* and *ST1* genes, and *E. coli* ITRC-18 [Industrial Toxicology Research Centre (ITRC), Lucknow, India] for *stx1, stx2, hlyA, eaeA,* and *chuA*.

### Determination of susceptibility to antimicrobials

We tested the isolates positive for virulence genes specific to EHEC from each site for susceptibility to 15 antimicrobials from six classes: aminoglycosides (amikacin, 10 μg/disc; gentamicin, 10 μg/disc; neomycin, 30 μg/disc; streptomycin, 10 μg/disc); β-lactams (piperacillin, 100 μg/disc; ampicillin, 10 μg/disc; amoxycillin, 30 μg/disc); cephalosporins (ceftazidime, 30 μg/disc; cephalothin, 30 μg/disc); folate inhibitors (co-trimoxazole, 25 μg/disc); fluoroquinolones (ciprofloxacin, 5 μg/disc; norfloxacin, 10 μg/disc); phenicols (chloramphenicol, 10 μg/disc); quinolones (nalidixic acid, 30 μg/disc); and tetracyclines (tetracycline, 30 μg/disc). These tests were performed as previously described (Ram et al. 2007) using an agar-diffusion method and antimicrobial impregnated paper discs (Hi-Media Ltd., Mumbai, India) as described by the Clinical and Laboratory Standards Institute (CLSI 2005). Each test was performed in triplicate for each *E. coli* isolate and antimicrobial. Data for susceptibility to antimicrobials tested for each bacterial isolate has been reported as resistant, intermediate (isolates with reduced susceptibility), and sensitive, based on Clinical and Laboratory Standards Institute break points (CLSI 2005).

### Statistical analyses

To analyze associations between responses of *E. coli* isolates for two antimicrobials and to assess the coselection (Seigal and Catellan 1987), we performed the Fisher’s exact test. We also used the Fisher’s exact test to determine significance of association between two genes. The significance level for all statistical analyses was assessed at α < 0.05.

## Results

### Quantitative enumeration of coliform populations

We found that the drinking water distribution system was contaminated by *E. coli* except at site 4 (Table 2). Before entering the water distribution system, potable water was microbiologically fit for drinking because the total coliform and fecal coliform count was zero. The maximum total coliform and fecal coliform populations were recorded at site 2, followed by site 3, site 6, and site 5. Drinking water pipelines at these sites had rust damage, which allowed seepage and thus contamination of the water.

### Occurrence of virulence genes specific to EHEC

Only 30% of the *E. coli* isolates (*n* = 60) screened were positive for virulence determinants of EHEC (Table 3). Our observations on virulence markers indicate that the potable water distribution system in Lucknow is contaminated by *E. coli* isolates (Table 3) exhibiting genes for expression of shiga toxins (33.3% of isolates exhibit both *stx1* and *stx2*, whereas 55.6% and 11.1% of isolates harbor the *stx2* or *stx1* gene, respectively). The *chuA* and *hlyA* genes were present in 16.7% and 23.3% of isolates, respectively. The occurrence of the *eaeA* gene was significantly (significant at the 5% level) associated with the *stx1* and *stx2* genes.

### Susceptibility to antimicrobials

All of the potential EHECs (*n* = 18) in the present study were resistant to at least one antimicrobial (Table 3). We found that 45.5% of isolates from site 2 were resistant to more than three antimicrobials. The resistance to cephalothin was significantly associated with nalidixic acid (significant at the 5% level). Similarly resistance to tetracycline was observed to be significantly associated with resistance to nalidixic acid (significant at the 5% level). Only one isolate from site 5 (IV1A) was resistant to ciprofloxacin. This isolate was also resistant to amoxycillin, cephalothin, nalidixic, and tetracycline and possessed reduced susceptibility to norfloxacin and piperacillin (Table 3). We found that 33.3% of isolates were resistant to tetra-cycline. Of the isolates we recovered, 27.7% exhibited resistance to the β-lactam class of antimicrobials. We found that 61.1, 38.9, and 11.1% of isolates possessed reduced susceptibility (intermediates) to neomycin, streptomycin, and piperacillin, indicating possible development of resistant strains in the future (Table 3).

## Discussion

Globally, > 1.1 billion people drink unsafe water. A vast majority of diarrheal diseases are attributable to unsafe water, sanitation, and hygiene [World Health Organization (WHO) 2002]. In India, a large population depends on processed surface waters for drinking. Analysis for fecal-indicator bacteria provides a sensitive, although not the most rapid, indication of pollution in drinking water supplies (WHO 2002). In the present study, we found potable water samples to be contaminated by coliform and fecal coliform bacteria. The level of *E. coli* or thermotolerant bacteria should be zero in a 100-mL sample of water directly intended for drinking or in treated water entering a distribution system (WHO 2002). However, certain drinking water standards allow the presence of 10 coliforms/100 mL in drinking water [Bureau of Indian Standards (BIS) 1991]. All of the positive samples in the present study exceeded the standard permissible limits recommended by various regulatory bodies for drinking water (BIS 1991; WHO 1993). The results suggest the possibility of contamination of percolating water distribution systems by fecal contaminants. We found that leaking sewage lines and human and animal excreta flowing into open drains are the most common potential sources of contamination in defective drinking water distribution systems. The *E. coli* we detected were in a culturable metabolic state. The plausible explanation could be recent contamination of the water distribution system by human and/or animal feces due to defective sewage lines and storage tanks. A similar situation was observed in the Netherlands in private drinking water supplies contaminated by STEC due to defective sewage lines (Schets et al. 2005).

*E. coli* isolates from human and cattle stool samples from India have been reported to exhibit *stx1* and *stx2* (Khan et al. 2002; Wani et al. 2006). The presence of the *eaeA* gene makes these isolates more virulent because this gene is required for expression of the full virulence of STEC in humans, leading to hemorrhagic colitis and hemolytic uremic syndrome (Boerlin et al. 1999). In the present study, the *E. coli* isolates positive for *stx1, stx2,* or both genes together also possess the *eaeA* gene. The *chuA* gene is part of heme transport locus that encodes for a 69-kDa outer membrane protein responsible for heme transport. The presence of the *chuA* gene, which imparts the ability to utilize heme or hemoglobin as an iron source, may enhance the virulent nature of certain isolates of EHEC that we recovered (Torres and Payne 1997). Therefore, a human population that consumes drinking water contaminated with the virulent form of EHEC is at high risk of hemorrhagic diarrhea.

In the present study, a plasmid-encoded gene (*hlyA*) responsible for hemolysin production was most prevalent among the isolates. It is likely that *E. coli* may transition between virulent and nonvirulent forms by acquiring or losing virulence genes encoded by plasmid(s), making these forms indistinguishable from normal gut flora in individuals consuming water for daily needs (Hall and Barlow 2004). The transformation of nonpathogenic form to pathogenic entity has been demonstrated for an enteric pathogen (*Vibrio cholerae*) that produces a phage-encoded enterotoxin (Faruque et al. 1998). Hence, human populations exposed to contaminated drinking water will enrich the environmental gene pool of *E. coli* that may serve as reservoirs of virulence determinant genes. In the present study, we observed resistance to multiple antimicrobials in *E. coli* recovered from potable water. Other studies have also found resistance to two or more antimicrobials in EHEC/STEC isolates from humans, surface waters, cattle, and food (Cergole-Novella et al. 2006; Manna et al. 2006; Ram and Shanker 2005). Also, Webster et al. (2004) reported that *E. coli* isolates from urban areas/point sources have resistance to more antimicrobials than rural/non–point source isolates, possibly because of greater exposure to antimicrobials. Some earlier studies have also shown that clinical and surface water isolates of *E. coli* that exhibited resistance to ciprofloxacin were multiantimicrobial resistant (Karlowsky et al. 2006; Ram and Shanker 2005; Ram et al. 2007). The resistance to nalidixic acid was significantly associated with cephalothin and tetracycline The significant statistical association observed between resistance to antimicrobials of two different classes was probably caused by coselection.

In the present study, a number of isolates exhibited resistance to tetracycline and the β-lactam class of antimicrobials. The resistance to these specific antimicrobials is sometimes encoded by plasmids, which may distribute resistance in susceptible bacteria through horizontal gene transfer (Hall and Barlow 2004; Sayah et al. 2005). Our findings indicate that the *E. coli* we recovered in this study express a high level of resistance to antimicrobials that are commonly used in clinical medicine (tetracycline, amoxycillin, ampicillin). This could contribute to the spread and persistence of antimicrobial-resistant bacteria and resistance determinants in humans and the environment. In most developing countries, diarrheal diseases are treated by an inadequate quantity of antimicrobials, without first identifying a pathogen. This is probably one of the most important factors for multiple antimicrobial resistances in potential EHEC isolates observed in the present study. Yoh and Honda (1997) reported that administration of antimicrobials in management of EHEC infections may cause disease progression by the release of shiga toxins *in vivo* through bacterial cell lysis, finally resulting in host death. Hence, the dissemination of resistance to antimicrobials among EHEC isolates may have potential negative clinical implications for therapeutic advancement, thus suggesting that antimicrobial therapy should be combined with the oral administration of shiga-toxin–binding or inactivating agents in EHEC infections (MacConnachie and Todd 2004). *E. coli* with reduced susceptibility (intermediates) for multiple antimicrobials in surface water and other environmental isolates have been reported (Ram et al. 2007; Sayah et al. 2005). Among drinking-water isolates, the emergence of resistance and decreasing levels of susceptibility (intermediates) of *E. coli* to a wide spectrum of antimicrobials is a matter of concern because it may limit the availability of antimicrobials for clinical management of waterborne outbreaks in the future.

The present study has certain limitations in statistical analysis of data due to limited sample size as well as unavailability of specific antimicrobial usage data of the residents in the area. However, the study does reveal the emergence of resistance and decreasing levels of susceptibility to antimicrobials in virulent isolates of EHEC recovered from potable water. Future studies intend to explore spatial and temporal variation in level of contamination caused by diarrheagenic *E. coli*.

## Conclusion

The presence of *E. coli* and fecal coliforms in potable water collected from a defective water distribution system impacted by leaking sewage lines and open drains may pose health risks to people using the domestic water supply for drinking and other domestic purposes. In spite of the small sample size, the results of the present study emphasize the human health risk associated with exposure to contaminated drinking water due to the presence of multi-antimicrobial-resistant *E. coli* exhibiting virulence genes specific to EHEC. Therefore, the presence of potential EHEC in drinking water distribution systems of developing nations requires increased surveillance for risk assessment and prevention strategies to protect public health.

## Figures and Tables

**Table 1:** PCR primers used in the amplification of virulence genes in drinking water isolates of *E. coli.*

Virulence gene	Primer sequence (5′–3′)	Product size (bp)
*stx1*	*stx1*: forward, CTGCCGGACACATAGAAGGAAACT	267
	*stx1*: reverse, AGAGGGGATTTCGTACAACACTGG	
*stx2*	*stx2*: forward, GGAGTTCAGTGGTAATACAATG	149
	*stx2*: reverse, GCGTCATCGTATACACAGG	
*eae A*	*eaeA*: forward, GAAGCCAAAGCGCACAAGACT	413
	*eaeA*: reverse, CTCCGCGGTTTTAGCAGACAC	
*hlyA*	*hlyA*: forward, GCTATGGGCCTGTTCTCCTCTGC	224
	*hlyA*: reverse, ACCACTTTCTTTCTCCCGACATCC	
*chuA*	*chuA*: forward, ATCGCGGCGTGCTGGTTCTTGTC	370
	*chuA*: reverse, TCGTCATTCGGCGCGGTTTCAC	

All the primers were previously described by Ram et al. (2007).

**Table 2:** Quantitative enumeration of coliforms in the drinking water distribution system.

	Most probable number/100 mL^a^	
Location	Total coliform	Fecal coliform
Site 1, Aishbagh Waterworks	ND	ND
Site 2, Charbagh Loco Thana	1,600	1,600
Site 3, Hussainganj	240	30
Site 4, Kaiserbagh	ND	ND
Site 5, Hazratganj	22	11
Site 6, Charbagh Railway Station	130	80
Control^b^	ND	ND

ND, none detected.

a Mean of three observations.

b Sterile Milli-Q water served as the control (Millipore, Bedford, MA, USA).

**Table 3:** Antimicrobial resistance and virulence determinants of potential EHEC in drinking water.

			Virulence genes^a^				
Location^b^	Isolate ID	Antimicrobial resistance^c^	*stx1*	*stx2*	*eaeA*	*hlyA*	*chuA*
Site 2	IB	Ch, Na (N, S, Ak)	+	−	+	−	−
	IC	Ch, Na, (N, S, T)	−	+	+	+	−
	1E	Ch (N, S)	+	−	+	+	+
	I2A	Na, T, Co (N, S)	+	+	+	+	+
	I2B	Ch (N, Cf, S)	−	+	+	+	−
	I2E	Ac, Ch, T, S, A, Pc, Co (N, Cf, S)	+	+	+	+	+
	I3A	Na (N)	−	+	+	+	−
	I3B	Ch, Na, T, Co	−	+	+	+	−
	I3C	Ch, Na	−	+	+	+	−
	I3D	Ac, Ch, Na, T, A, Pc	+	+	+	+	+
	I3E	Ac, Ch, Na, T, A, Pc	+	+	+	−	+
Site 3	IIA	Ch, A, Pc	−	+	+	−	−
Site 5	IV1 A	Ac, Ch, Na, T, Cf (Nx, Pc)	+	+	+	+	−
	IV3 A	Ch, (N)	+	+	+	−	+
	IVB	(Pc)	−	+	+	+	+
	IVG	Ch (N, S)	−	+	+	+	+
Site 6	VB	Ch (N)	−	+	+	+	+
	VE1	Ch (N, S)	−	+	+	+	+

A, ampicillin; Ac, amoxycillin; AK, amikacin; Cf, ciprofloxacin; Ch, cephalothin; Co, co-trimoxazole; ID, identification; N, neomycin; Na, nalidixic acid; Nx, norfloxacin; Pc, piperacillin; S, streptomycin; T, tetracycline. Isolates that possessed reduced susceptibility (intermediates) to antimicrobials are shown in parentheses.

a Positive controls: *E. coli* ATCC-43887 (*eaeA*) and *E. coli* ITRC-18 (*stx1, stx2, hlyA,* and *chuA*).

b Site 2, Charbagh Loco Thana; site 3, Hussainganj; site 5, Hazaratganj; site 6, Charbagh Railway Station.

c *E. coli* ATCC-25922 (American Type Culture Collection, Manassas, VA) was used as negative control in each experimental set, and the positive control was *E. coli* ITRC #GIG (sensitive to norfloxacin).
